# Effectiveness of typodont, quail egg and virtual simulation for ultrasonic periodontal scaling teaching among pre-clinical students: a randomized trial

**DOI:** 10.1186/s12903-023-03767-5

**Published:** 2024-01-16

**Authors:** Jiarun Fu, Zhentao Lao, Li Gao, Shiwen Wu, Xin Huang, Chuanjiang Zhao, Panpan Wang

**Affiliations:** 1 Hospital of Stomatology, Sun Yat-Sen University, No.55 Linyuan Xi Road, Guangzhou, Guangdong 510055 China; 2 Department of Periodontology, Hospital of Stomatology, Sun Yat-sen University, Guangzhou, Guangdong 510055 China

**Keywords:** Ultrasonic periodontal scaling, Pre-clinical education, Teaching effect, Virtual simulation, Typodont, Quail egg, Integrated teaching

## Abstract

**Background:**

This study aimed to compare the efficacy of three different techniques, namely virtual simulation technology (VS), traditional pathological typodont (TT), and quail egg (QE), in pre-clinical training of periodontal ultrasonic scaling. It also aimed to propose an integrated teaching approach for ultrasonic scaling teaching.

**Methods:**

This single-blind randomized multi-arm trial enrolled 108 fourth-year students from Guanghua School of Stomatology at Sun Yat-sen University. The participants were randomly, evenly assigned to VS, TT, or QE group. First, the participants received theoretical review on ultrasonic scaling and demonstrative teaching. Then in the 90-minute operation training by group, students used traditional typodont equipped in head-simulators, raw quail eggs, or scaling module of the UniDental VS system respectively. Then all participants practiced on pathological models for 30 min. In the final operation examination, participants were instructed to remove the supra- and sub-gingival calculi pre-set on designated teeth by ultrasonic scalers within 30 min. Their performances were evaluated by residual calculus rate and a multi-perspective scoring scale. After the examination, questionnaires were provided to assess the teaching effects of each method and the fidelity of VS. Statistical analysis was carried out using one-way, two-way ANOVA, and multiple t-test.

**Results:**

Students in VS group had significant higher total test scores than QE group (87.89 ± 6.81, 83.53 ± 8.14) and TT group (85.03 ± 6.81). VS group scored higher in several dimensional comparisons with the other two groups, especially in difficult situations. QE group had higher scores particularly in force application and supra-gingival scaling. TT group scored the highest in pivot stability practice and body position training. Students gave higher scores when assessing the fidelity of VS than experienced teachers.

**Conclusion:**

The study highlights the importance of specialized pre-clinical training on ultrasonic scaling for dental students. The methods adopted in current study (VS, TT and QE) each offered unique advantages in education, which can be combined to create an integrative teaching procedure. This procedure aims to provide an effective, advisable and normative pre-clinical training procedure for ultrasonic scaling. By utilizing the strengths of each method, dental educators can deliver high-quality training and ensure that students are well-prepared for clinical practice.

**Supplementary Information:**

The online version contains supplementary material available at 10.1186/s12903-023-03767-5.

## Background

Scaling and root planing (SRP) is a common non-surgical periodontal therapy that involves removing dental plaque and calculus, then smoothing the root surfaces using ultrasonic or manual scalers [1]. It is an effective and necessary procedure for treating periodontitis in outpatients [2, 3]. However, long period of repetitive hand motions, high pinch force and sustained hand postures often cause work-related musculoskeletal diseases and pain among dental professionals [4]. The prevalence of these conditions varies from 10.8 to 97.9% [5].

The ultrasonic scaler is made up of an ultrasonic wave generator and a transducer handset. It converts high frequency electric energy into ultrasonic vibration (18000-50000 Hz) to disrupt bacteria biofilm and calculus more efficiently, which can then be flushed away from the teeth by a small jet of water emitted from the top of the scaler [2]. The use of shock waves combined with the water jet could save 20–50% time during SRP procedures, reduce clinical fatigue for dentists, and increase their tactile sensitivity over time [6–8]. In addition, repeated use of manual scaling may cause uncontrollable cementum loss due to sharp-edged work tip, while ultrasonic therapy, using high-frequency mechanical force, void effect, micro-flow force, and flushing action, has been shown to significantly reduce the cementum damage [7, 9, 10]. Due to these advantages, the ultrasonic scaler has become a widely accepted instrument in clinics. A recent practice-based study showed that the application frequency of both manual and ultrasonic scaler was similar in periodontal therapy, with 94.4% of periodontists combining both methods [11].

Correct hand feel during SRP is crucial, especially when working inside periodontal pockets where direct vision is not available. Ultrasonic scalers require even more precise efforts to make their distinct shockwave and void effect work effectively. A proper ultrasonic scaling hand feel involves explicit lateral pressure, as well as proper sliding force and movement of the scalers. However, the ultrasonic vibration of the scaler can make it difficult to catch such subtle movements, which increases susceptibility to technology. To overcome this difficulty, specialized systemic tutoring and repeated live-action training are necessary during pre-clinical practice.

Unfortunately, many dental schools lack such specialized training on ultrasonic scaling [12]. The reasons include limited teaching time, the current curricula outline setting focused on manual instruments, the relative shortage of effective laboratory resources, and the infeasibility of repeated intrusive training on patients [13]. To address these deficiencies, some dental schools have introduced several traditional materials, such as quail eggs, pop-top cans, and bionic models of extracted teeth, into pre-clinical periodontal education [14–17].

Virtual simulation (VS) technology may be a real boon in such training [13, 18–20]. With multi-sensory feedback capabilities, VS can create highly realistic specialized scaling environment, provide real-time standardized assessment, and offer repeatable practicing opportunities for students. This allows for sustainable self-learning [21, 22]. While the effect of VS technology in ultrasonic scaling teaching has not been reported yet, it is an area of potential future development that could greatly enhance pre-clinical education for dental students.

To explore new effective ways which may promote the reform of ultrasonic scaling teaching, effectiveness of quail egg, VS system assisted ultrasonic training were compared with traditional pathological typodont scaling in this randomized trial. Through comprehensive analysis of laboratory results, this study aimed to propose an integrative teaching procedure which could synthesize strengths of each method. Such a method could provide an effective, advisable and normative pre-clinical training procedure for ultrasonic scaling.

## Methods

### Participants

108 fourth-year students from Guanghua School of Stomatology at Sun Yat-sen University were enrolled in this multi-arm randomized trial. Participants were included according to the following criteria: (1) Fourth-year undergraduates. (2) On the pre-clinical stage. (3) Finish the theoretical course of Periodontology. (4) Volunteer to participate. Participants who could not finish the whole trial were excluded. The whole process was carried out under the protocol approved by Medical Ethics Committee of Hospital of Stomatology, Sun Yat-sen University (KQEC-2023-06-01) and was registered in Chinese Clinical Trial Registry (ChiCTR2300071647, Reg Date: 22/05/2023). The study established a significant level of α at 0.05, a statistical power of test (1-β) of 0.90, and an effect size (*f* = 0.52) based on means and standard deviation (SD) of preliminary experiments. The sample size of this study estimated by the software G*Power [23](version 3.1.9.7) was 51 (Supplementary Fig. 1). Therefore, the study recruited over 51 participants, ensuring adequate population representation.

### Experimental materials

**Fig. 1:**
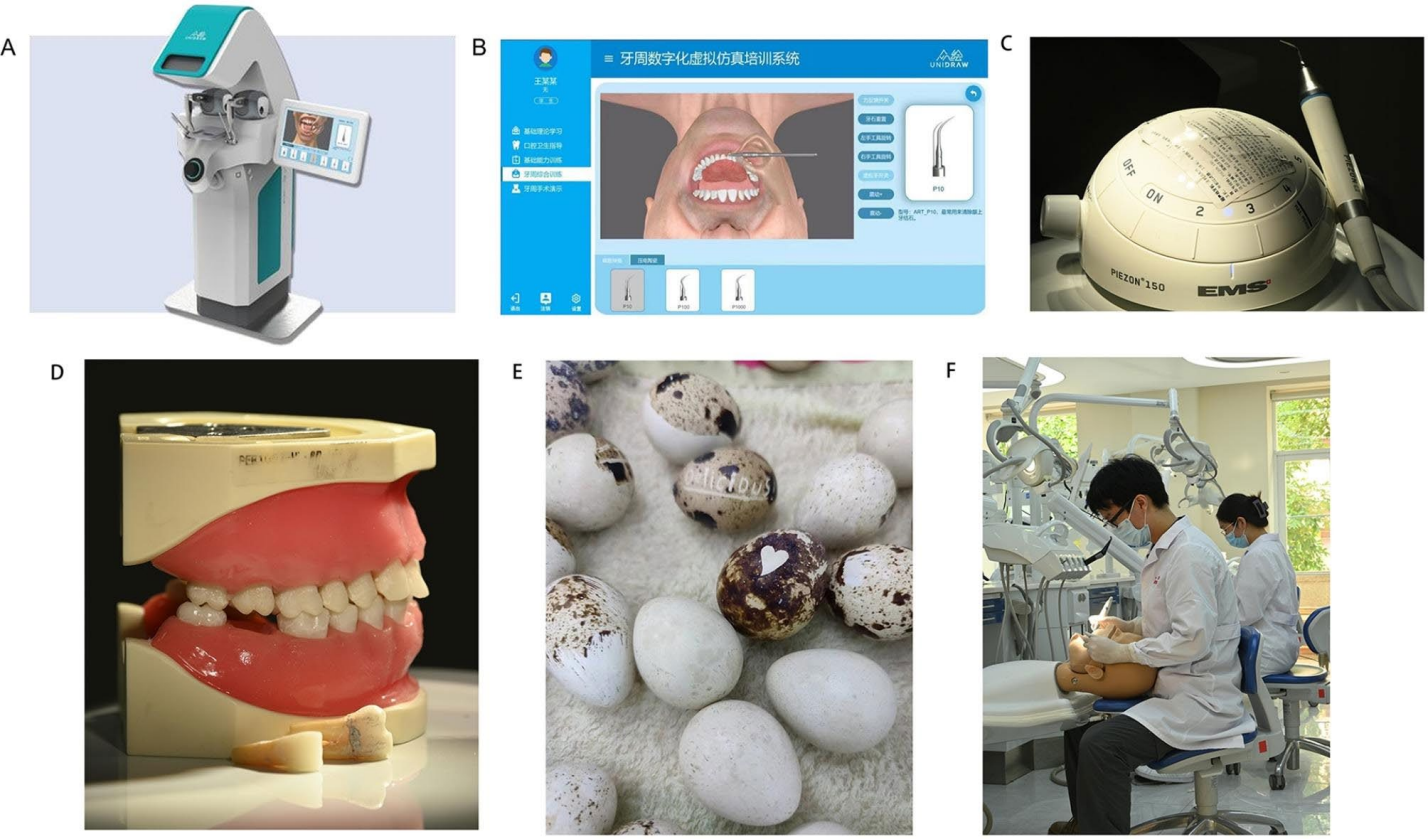
Illustration of the three ultrasonic scaling training methods. (**A**) Overview of UniDental VS system. (**B**) Main functional interface of the UniDental Dental simulator, which contains 5 parts: instrument selection, position alternation, simulative scaling, real-time feedback and calculi reset. (**C**) The ultrasonic wave generator and handset with the work tip used in the current study. (**D**) Traditional periodontal pathological typodont and residual calculi observed on the removable teeth. (**E**) Quail eggs for scraping practice. (**F**) Photos captured during the training program. Students were practicing ultrasonic scaling on the traditional typodont equipped in head-simulators

In this experiment, UniDental VS system (Fig. 1A-B), ultrasonic handsets (HD-7 H, EMS, Swiss), supra-gingival work tips (PIEZON-P, EMS, Swiss), sub-gingival work tips (PIEZON-P, PS, EMS, Swiss), ultrasonic wave generators (PIEZON 150, EMS, Swiss) (Fig. 1C), periodontal pathological typodonts (Nissin Dental Products Inc., Japan) (Fig. 1D), calculus sets (Nissin Dental Products Inc., Japan), and quail eggs (Fig. 1E) were used.

The UniDental VS system was developed by Beijing UniDraw Virtual Reality Technology Research Institute Co., Ltd. in 2014 and has been utilized in 40 Chinese dental schools. Its primary function is to generate a virtual reality environment for the purposes of basic teaching of stomatology, dental skills training, and assessment. It creates a virtual environment that includes a virtual maxillofacial model, dentition and tools, which are displayed using computer imaging technology. Through its 6-degree of freedom 3-dimensional force feedback mechanism, the system can offer real-time (1000 Hz) accurate and detailed force feedback to meet the demands to realize ultrasonic scaling simulation. The force feedback mechanism is accessible through a button that, when activated, transfers mimetic forcible feedback to students through handset.

### Study procedure

#### Randomization and blinding

The teaching secretary randomly assigned 108 eligible students to VS group, traditional pathological typodont (TT) group and quail egg (QE) group. The assignment was based on Student ID using a random number table, with 36 students allocated to each group. All the students were not informed of the purpose of the experiment. To avoid bias in the final examination scores, the teachers responsible for grading the students were unaware of their group assignments.

#### Theoretical and demonstrative teaching

**Fig. 2:**
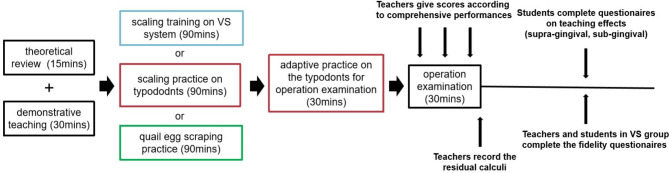
A brief flowchart of the current study

All students uniformly received a 15-minute theoretical review via lantern slides, followed by a 30-minute demonstration and teaching of scaling on a periodontal pathological typodont by a senior periodontist (Fig. 2).

#### Operation training

The participants underwent a 90-minute hands-on training session separately in their respective groups, with itinerant teaching provided by teachers to answer questions and correct students’ postures. All groups shared the same teachers. In TT group, students conducted scaling training using periodontal pathological typodonts equipped in head-simulators. Students in QE group were given a raw quail egg and asked to use ultrasonic scalers to scrape off all patterns on the eggshell without breaking it. Students in VS group practiced using the periodontal scaling module of the UniDental VS system, which contains 5 parts: instrument selection, position alternation, simulative scaling, real-time feedback and calculi reset (Fig. 1B). Students should get the scaler and put it into the correct place in the right body position or a warning would be sent. The system displayed calculi in perspective, and only when the correct scaling force, angle, lateral pressure, and movement were applied to the dental surfaces would the calculi be removed. During the VS course, students could request help from teachers online.

Following the training sessions, all participants practiced their skills and adapted to the test environment on pathological models for an additional 30 min.

#### Operation examination

In the operational examination, 108 students were tasked with scaling off all calculi on the six designated teeth (11, 31, 16, 17, 36, and 47), which had been preset on every surface (mesial, distal, buccal, lingual), distributed across supra-gingival and sub-gingival areas within a 30-minute time limit. After the completion of the operation, the typodonts were thoroughly examined by the teachers to identify any residual calculi at specific sites. Subsequently, the students’ comprehensive performances were assessed based on the established multi-perspective scoring scale, which included evaluations of dental tool selection, body position, periodontal probing, tool gripping, firm pivot, angle of worktip, generation of strength, direction of force, movement magnitude, checking with probe, calculus removal, and avoiding soft tissue injury (Supplementary Table 1).

### Questionnaire survey

After the examination, participants were given self-designed questionnaires to gather their thoughts and feelings regarding the teaching effects. The questionnaires contained items assessing whether the group trail really helped them in maintaining proper body position, mastering the proper grip of tools, approaches of work tips, ways and angles to fit work tips with tooth surfaces, lateral pressure, movement and sliding force, pivots and sequence and consistency of scaling, as well as improving scaling efficiency and reducing injuries to tissues during scaling. Each student completed a supra-gingival questionnaire and a sub-gingival questionnaire (Supplementary Tables 2 and Supplementary Table 3). Additionally, another questionnaire evaluating the fidelity of the VS system was distributed to 36 students in VS group as well as 5 teachers (Table 1). The response options for each item on all questionnaires included: 1 = strongly dissatisfied or strongly disagree, 2 = dissatisfied or disagree, 3 = neutral, 4 = satisfied or agree, and 5 = very satisfied or strongly agree. After collecting the questionnaires, the average score of each item was calculated.

**Table 1:** Evaluation of VS fidelity by students and teachers

		Students (N = 36)	Teachers (N = 5)	*P* value
Shape and color of teeth, gingiva and calculus		3.94 ± 0.98	3.00 ± 0.71	0.046*
Shape and color of dental tools		4.25 ± 0.73	3.60 ± 0.55	0.064
Shape and color of oral environment		4.06 ± 0.89	3.00 ± 0.71	0.016*
Magnitude and direction of force		3.86 ±0.80	2.60 ± 0.55	0.002**
Allowable moving and orientation range of worktip		3.86 ± 1.05	2.00 ± 0.71	< 0.001***
Stiffness and friction of teeth and gingiva		4.06 ± 0.79	2.40 ± 0.89	< 0.001***
Feeling of splitting calculus from dental faces		3.86 ± 0.96	2.40 ± 0.89	0.003**
Fidelity of response of virtual patients		3.83 ± 0.85	2.40 ± 0.89	0.001**

Statistical analysis was performed using multiple t-test, N(students) = 36, N(teachers) = 5, *P < 0.05, **P < 0.01, ***P < 0.001

### Statistical analysis

**Fig. 3:**
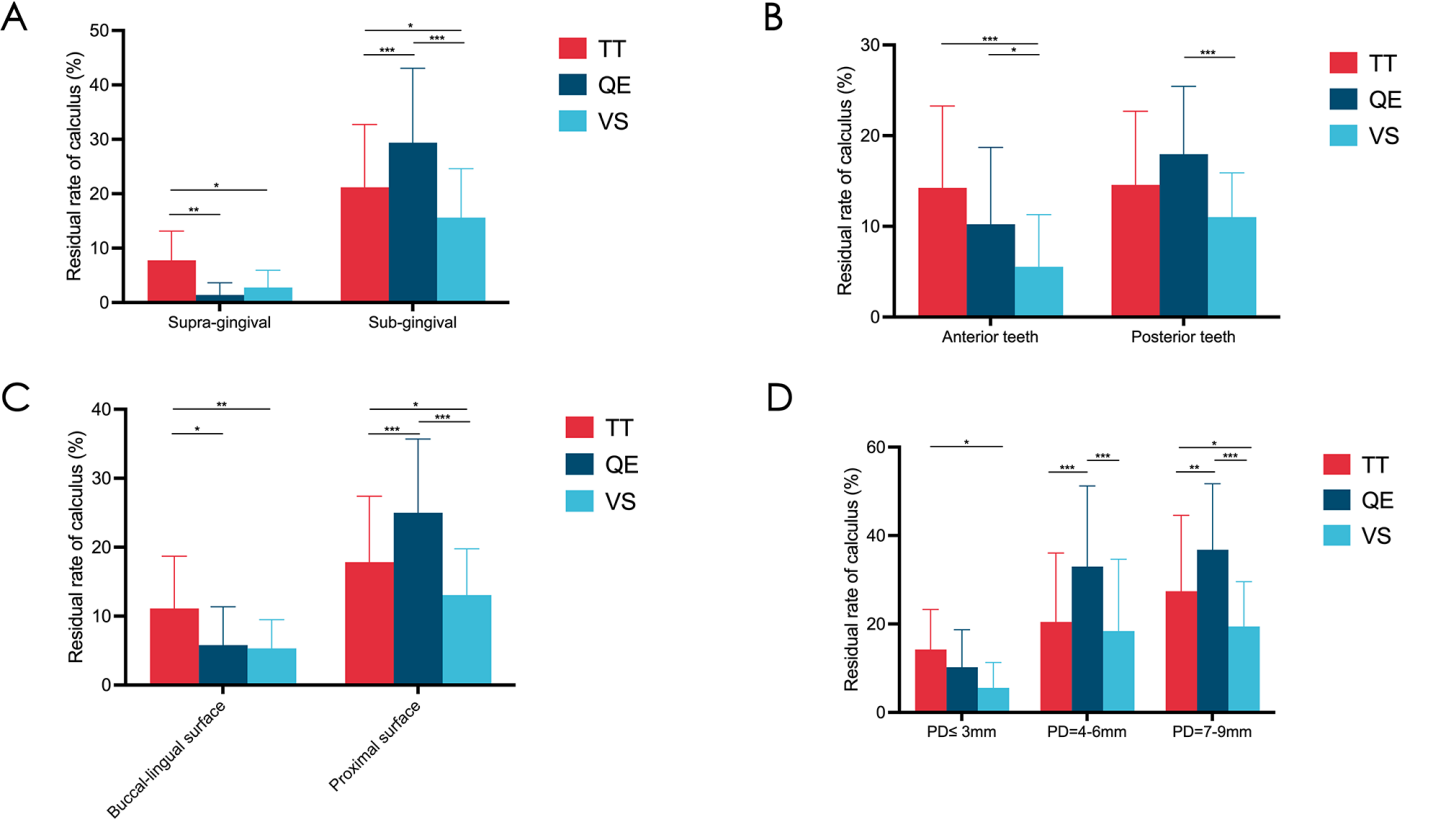
Comparison of residual calculus rates of the three groups. (**A**) Residual calculus rate of the supra-gingival was significantly lower than that of the sub-gingival (^***^P < 0.001). TT group had the most residual calculi at supra-gingival, while QE group had the most at sub-gingival. (**B**) Residual calculus rate of anterior teeth was significantly lower than that of the posterior (^***^P < 0.001). VS group had the least residual calculi in anterior teeth. QE group had obviously more calculi left than VS group in the posterior. (**C**) Residual calculus rate of buccal-lingual surfaces was significantly lower than the proximal (^***^P < 0.001). TT group had the most residual calculi on buccal-lingual surfaces, while QE group had the most on proximal surfaces. (**D**) Residual calculus rates increased with pocket depth (^***^P < 0.001). TT group had the most residual calculi in shallow pockets, while QE group had the most in medium and deep pockets. (Statistical analysis was performed using two-way ANOVA, N = 108, ^*^P < 0.05, ^**^P < 0.01, ^***^P < 0.001)

**Fig. 4:**
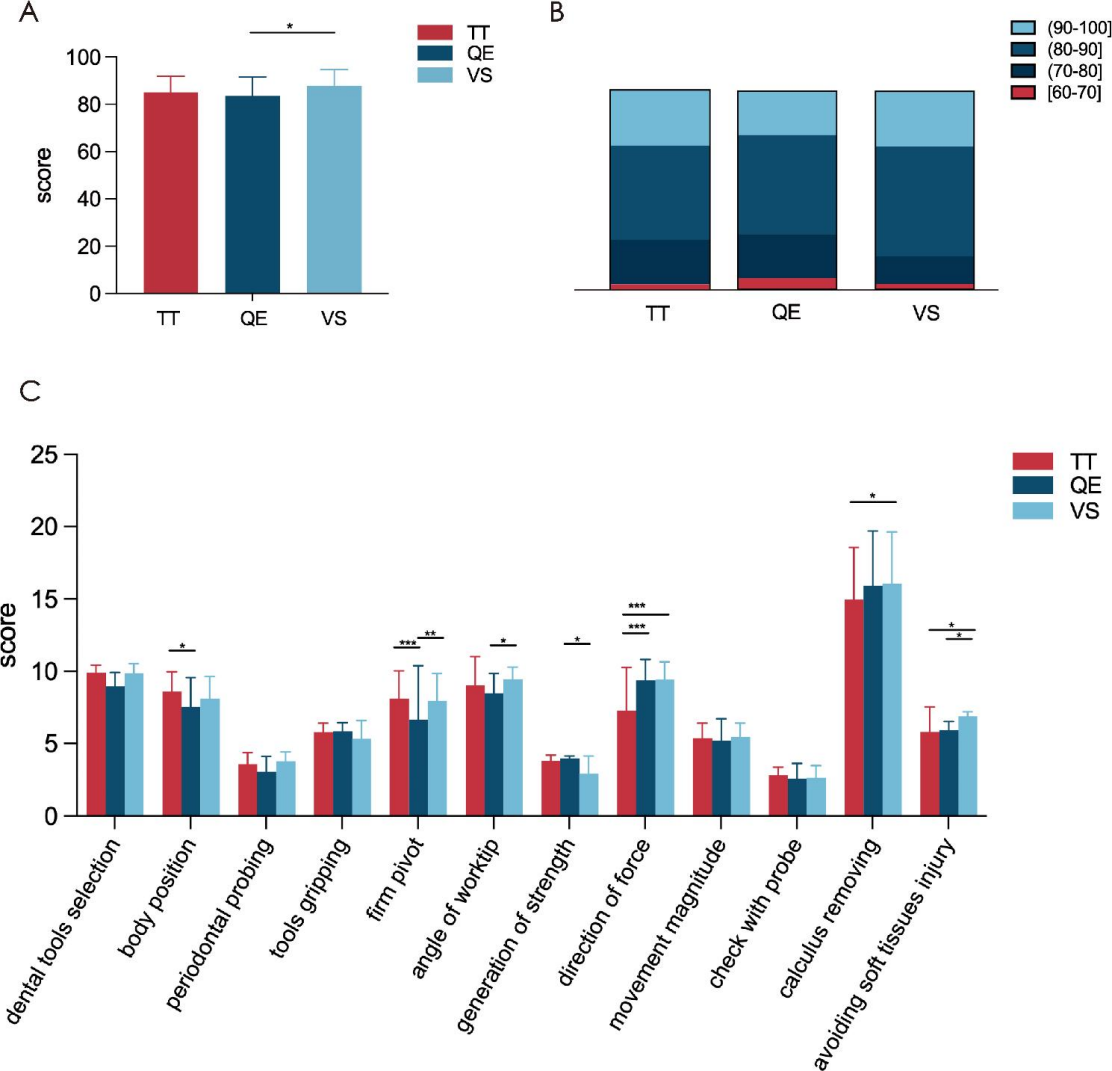
Comparison of the test scores of students in the three groups. (**A**) Comparison of the total scores of three groups. VS group scored significantly higher than QE group in total. (Statistical analysis was performed using one-way ANOVA, N = 108, ^*^P < 0.05, ^**^P < 0.01, ^***^P < 0.001). (**B**) Stacked bar chart showing the distribution of total test scores among the three groups. (**C**) Comparison of the specific scores in different test dimensions of three groups. (Statistical analysis was performed using two-way ANOVA)

**Fig. 5:**
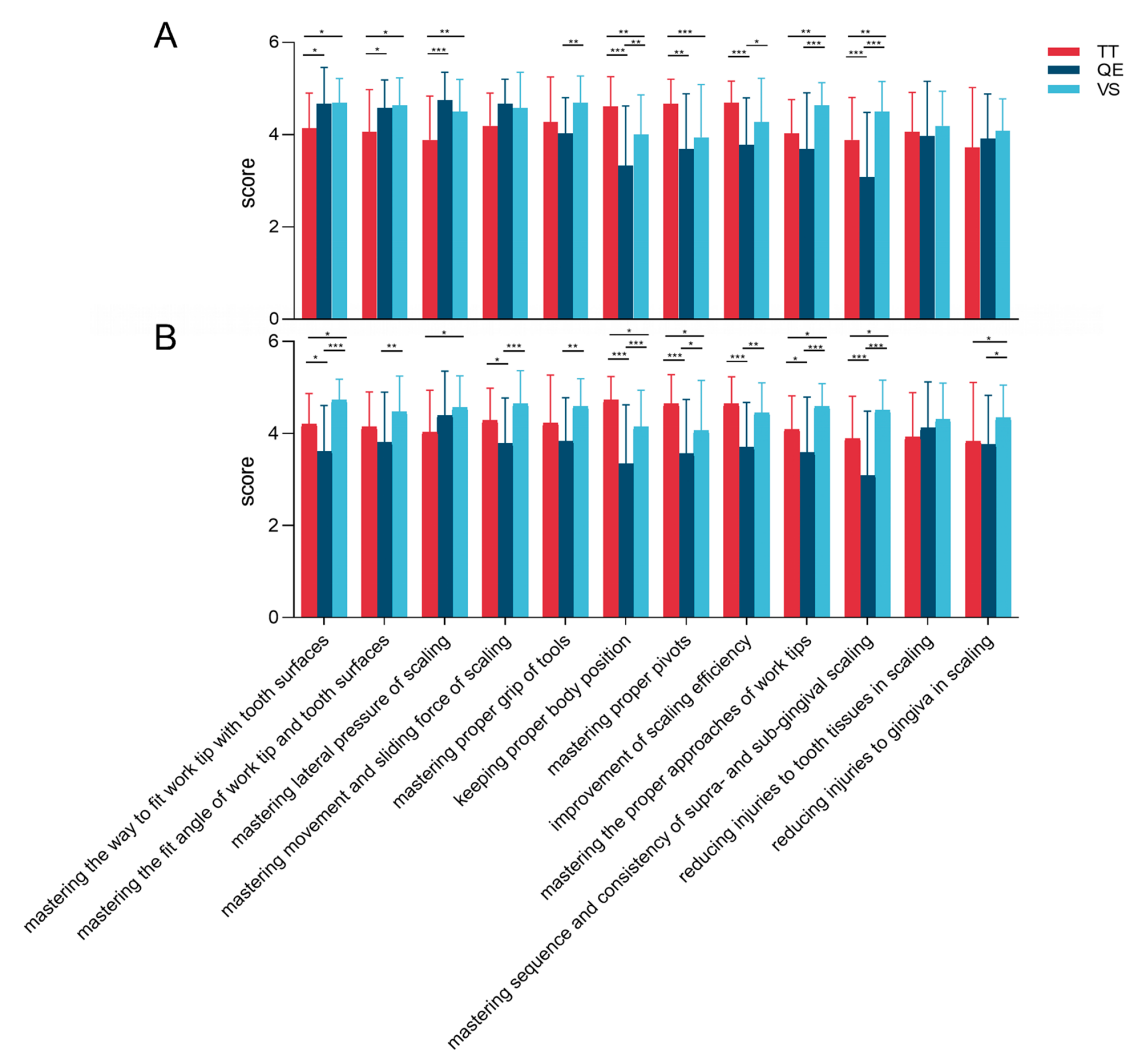
Comparison of the teaching effects in the three groups. (**A**) Supra-gingival; (**B**) Sub-gingival. (Statistical analysis was performed using two-way ANOVA, N = 108, ^*^P < 0.05, ^**^P < 0.01, ^***^P < 0.001)

Data analysis was conducted using SPSS 20 statistical software (IBM Inc., Chicago, IL). The calculus-scaling performance was assessed by residual calculus rate at the corresponding site in percentage form. The residual calculus rate, final exam scores, assessment of teaching effects and VS fidelity were reported as mean ± SD. Statistical analysis was carried out through two-way ANOVA (Figs. 3A-D, 4C and 5), one-way ANOVA (Table 2; Fig. 4A), and multiple t test (Table 1). *P* value less than 0.05 was considered statistically significant.

**Table 2:** Demographic characteristics of the three groups

	TT	QE	VS
Age	22.00 ± 0.34	22.08 ± 0.28	22.06 ± 0.33
Gender (female/male)	23/13	21/15	23/13
Class distribution (5-year/ "5 + 3" program)	20/16	20/16	20/16
Average grade-point	3.412 ± 0.58	3.390 ± 0.58	3.488 ± 0.56

Statistical analysis was performed using one-way ANOVA, N = 108, *P* < 0.05 was considered as statistically significant

## Results

### Demographic characteristic and ordinary performance

Ages and average grade-points of participants were reported as mean ± SD, while gender and class distribution were presented as ratios. The analysis revealed no significant inter-group differences in age, gender, class distribution or ordinary major performance (Average Grade-Point) (Table 2).

### Comparison of the residual calculus rates

All participants completed the operation examination as requested, and a total of 5184 scaled tooth surfaces were examined and recorded. The residual calculus rates were analyzed based on calculi location, tooth position and periodontal pocket depth.

More calculi were left in sub-gingival pockets than supra-gingival ones (22.07%±6.93% vs. 3.97%±3.35%, ^***^P < 0.001). The residual rates of supra-gingival calculi in the QE and VS group were significantly lower than that of TT group (1.39%±2.23% vs. 7.76%±5.38%, ^**^P = 0.006) (2.78%±3.15% vs. 7.76%±5.38, ^*^P = 0.040) (Fig. 3A). As for the sub-gingival calculi, the residual rate of QE group (29.40%±13.66%) was significantly higher than that of the other two groups (^***^P < 0.001), and TT group was slightly higher than the VS group (21.18%±11.55% vs. 15.63%±8.99%, ^*^P = 0.018).

There were more overall calculi remaining in posterior teeth than in anterior teeth (14.53%±3.47% vs. 10.01%±4.35%, ^***^P < 0.001). The VS group had a lower residual rate both in anterior and posterior teeth. It had the least number of calculi left in anterior teeth (VS vs. TT: 5.56%±5.74% vs. 14.24%±9.04%, ^***^P < 0.001) (VS vs. QE: 5.56%±5.74% vs. 10.24%±8.47%, ^*^P = 0.022), and had a significantly lower residual rate than QE group in posterior teeth (11.02%±4.87% vs. 17.97%±7.48%, ^***^P < 0.001).

Residual calculus in proximal areas exceeded that in buccal-lingual regions. (18.63%±6.00% vs. 7.41%±3.22%, ^***^P < 0.001). The TT group had more residual calculi on buccal-lingual surfaces than the other two groups (TT vs. QE: 11.11%±7.59% vs. 5.79%±5.57%, ^*^P = 0.011) (TT vs. VS: 11.11%±7.59% vs. 5.32%±4.18%, ^**^P = 0.005). While in terms of the calculi attached to proximal surfaces, the residual rate of QE group (25.00%±10.68%) was significantly higher than the other two groups (^***^P < 0.001), and TT group was slightly higher than the VS group (17.82%±9.59% vs. 13.08%±6.69%, ^*^P = 0.026).

The calculi residual rate was significantly lower in shallow periodontal pockets (probing depth (PD) ≤ 3 mm, 10.01 ± 4.35%) than in medium pockets (PD = 4-6 mm, 23.96 ± 7.89%, ^***^P < 0.001) and deep ones (PD = 7-9 mm, 27.89 ± 8.69%, ^***^P < 0.001). QE group had obviously more calculi left than TT and VS group in medium (QE vs. TT: 32.99%±18.21% vs. 20.49%±15.57%, ^***^P < 0.001) (QE vs. VS: 32.99%±18.21% vs. 18.40%±16.23%, ^***^P < 0.001) and deep pockets (QE vs. TT: 36.81%±14.92% vs. 27.43%±17.12%, ^**^P < 0.010) (QE vs. VS: 36.81%±14.92% vs. 19.44%±10.11%, ^***^P < 0.001). VS group had fewer residual calculi than TT group in shallow (5.56%±5.74% vs. 14.24%±9.04%, ^*^P = 0.019) and deep pockets (5.56%±5.74% vs. 27.43%±17.12%, ^*^P = 0.034).

### Comparison of the operation test scores

In total, 108 scoring sheets of participants were summarized and analyzed. The mean total examination score in the VS group was significantly higher than QE group (87.89 ± 6.81 vs. 83.53 ± 8.14, ^*^P = 0.033), while the TT group (85.03 ± 6.81) performed in between (Fig. 4A). The stacked bar chart illustrated that the distribution of scores in the VS group was less polarized (Fig. 4B). As for the specific dimension-based results in examination (Fig. 4C), QE group scored significantly higher in “generation of strength” than VS group (3.97 ± 0.17 vs. 2.92 ± 1.23, ^*^P = 0.025). QE and VS group both scored higher than the TT group in “direction of force” (^***^P < 0.001, ^***^P < 0.001). TT group showed better results than the QE group in “body position” (8.58 ± 1.38 vs. 7.56 ± 2.02, ^*^P = 0.031). TT group also scored higher than QE group in “firm pivots” (^***^P < 0.001), and VS group was just as good (^**^P = 0.003). VS group showed absolute higher scores than TT and QE group in “avoiding soft tissues injury” (^*^P = 0.021, ^*^P = 0.044), and scored higher in “angle of work tip” than QE group (9.44 ± 0.84 vs. 8.47 ± 1.38, ^*^P = 0.044), and in “calculus removing” than TT group (16.06 ± 3.57 vs. 14.97 ± 3.61, ^*^P = 0.021).

### Teaching effects assessment results from the questionnaire

A total of 108 questionnaires were distributed, and the response rate was 100%. Results of the analysis showed that (Fig. 5) both QE and VS group had higher scores than the TT group in mastering various aspects of supra-gingival scaling such as “the way to fit work tip with tooth surfaces” (^*^P = 0.028, ^*^P = 0.019), “the fit angle of work tip and tooth surfaces” (^*^P = 0.028, ^*^P = 0.013) and “lateral pressure of scaling” (^***^P < 0.001, ^**^P = 0.008).

However, it was not the case for sub-gingival scaling. In sub-gingival scaling, VS group scored the highest in “mastering the way to fit work tip” (^*^P = 0.035, ^***^P < 0.001), and TT group ranked second highest, with scores significantly higher than QE group (^*^P = 0.017). VS group scored higher than QE group in “mastering the fit angle” (^**^P = 0.005), and TT group in “mastering lateral pressure” (^*^P = 0.035). In addition, VS group had significant higher scores in “mastering movement and sliding force of scaling” and “reducing injuries to gingiva in scaling”. However, these results were observed to be not significant in supra-gingival scaling. To be specific, VS and TT group scored higher than QE group in “mastering the movement and sliding force of sub-gingival scaling” (^***^P < 0.001, ^*^P = 0.049), and VS group scored the highest in “reducing gingiva injuries” (^*^P = 0.049, ^*^P = 0.017).

There were also some interesting common findings between supra- and sub-gingival scaling. Specifically, TT group showed consistent higher scores than both the QE and VS group in “keeping proper body position” (supra-gingival: ^***^P < 0.001, ^**^P = 0.008) (sub-gingival: ^***^P < 0.001, ^*^P = 0.017) and “mastering proper pivots” (supra-gingival: ^***^P < 0.001, ^**^P = 0.001) (sub-gingival: ^***^P < 0.001, ^*^P = 0.017) for both categories of scaling. Additionally, TT and VS group both demonstrated consistent higher scores than the QE group in “improvement of scaling efficiency” both for supra- and sub-gingival scaling (supra-gingival: ^***^P < 0.001, ^*^P = 0.040) (sub-gingival: ^***^P < 0.001, ^**^P = 0.001). VS group consistently scored the highest among the 3 groups throughout scaling in terms of “mastering the proper approaches of work tips” (supra-gingival: ^**^P = 0.008, ^***^P < 0.001) (sub-gingival: ^*^P = 0.049, ^***^P < 0.001) and “mastering sequence and consistency of supra- and sub-gingival scaling” (supra-gingival: ^**^P = 0.008, ^***^P < 0.001) (sub-gingival: ^*^P = 0.011, ^***^P < 0.001). It also scored consistently higher than QE group in “mastering proper grip of tools” (^**^P = 0.003, ^**^P = 0.001).

### Evaluation of VS fidelity by students and teachers

A total of 41 questionnaires (36 students and 5 teachers) were distributed, and the response rate was 100%. Teachers gave significant lower scores than students in several areas such as “shape and color of teeth, gingiva and calculus” (3.00 ± 0.71 vs. 3.94 ± 0.98, ^*^P = 0.046), “shape and color of oral environment” (3.00 ± 0.71 vs. 4.06 ± 0.89, ^*^P = 0.016), “magnitude and direction of force” (2.60 ± 0.55 vs. 3.86 ± 0.80, ^**^P = 0.002), “allowable moving range and orientation range of work tips” (2.00 ± 0.71 vs. 3.86 ± 1.05, ^***^P < 0.001), “stiffness and friction of teeth and gingiva” (2.40 ± 0.89 vs. 4.06 ± 0.79, ^***^P < 0.001), “feeling of splitting calculus from dental surfaces” (2.40 ± 0.89 vs. 3.86 ± 0.96, ^**^P = 0.003) and “fidelity of response of virtual patients” (2.40 ± 0.89 vs. 3.83 ± 0.85, ^**^P = 0.001). However, no significant difference was found for “shape and color of dental tools” (*P* = 0.064).

## Discussion

Specialized pre-clinical training on ultrasonic scaling is crucial for dental students, and new teaching methods such as VS are urgently required to address teaching insufficiency. The primary objective of the current study was to compare the effectiveness of traditional typodont with quail egg and VS-assisted training in laboratory training of periodontal ultrasonic scaling skills for pre-clinical students. Additionally, the study sought to explore the fidelity of VS system, identify the relative merits of the 3 approaches, and propose an integrative teaching procedure to synthesize the strengths of each method. This approach aims to better promote and normalize students’ scaling skills in the periodontal pre-clinical teaching unit.

The results of the study indicated that teaching using VS technology improved calculus removal effects in most cases, particularly in difficult locations such as subgingival regions, proximal surfaces and deeper pockets. Moreover, it helped students to effectively master periodontal scaling techniques. However, it was found that VS was not a one-size-fits-all solution, as the QE or TT groups had better performance in certain areas. QE group demonstrated remarkable advantages in force control training and supra-gingival scaling training, while typodont was deemed the most suitable for body position and pivots training.

The use of quail eggs for subgingival ultrasonic scaling was first reported in 2016 [17], and the current study found that it offered teaching advantages in force application and supra-gingival scaling. This may be due to the fragility of eggs, which limited the range of scaling force and encouraged students to find the proper force that made the best use of ultrasonic vibration, rather than relying on brute force. The curvature of the egg surface was similar to that of teeth, making it easier for students to fit the work tip to the teeth’s surfaces. Additionally, the easy visibility of patterns removal results made the egg beneficial for the supragingival scaling procedures, as students could purposefully and voluntarily adjust the force for higher scaling efficiency during self-training.

Typodont was found to be prominent in pivot stability practice and body position training, and worked as well as VS in improving scaling efficiency. This was not surprising since pivot practicing on typodonts was the closest to reality. The pivots in the VS system were not flexible enough, which may have contributed to their lower performance in this area. Meanwhile, QE group also had distinct imperfections in pivot control training.

Considering both objective assessment indicators and questionnaire items, the VS system was found to be the most helpful teaching method. It showed absolute strengths in avoiding subgingival injuries, mastering the proper approaches of work tips, way to fit work tip as well as sequence and consistency of supra- and sub-gingival scaling. Students in VS group performed particularly well in subgingival teaching, with better mastery of the fit angle of work tip, movement and sliding force than the QE group, and more appropriate lateral pressure than the TT group. The program setting of VS contributed to good guidance for the proper fit between the work tip and tooth surface, because once the fit was not in place or fit angle was wrong, then simulated calculi would not be successfully removed, which greatly enhanced the effectiveness and accuracy of training. TT group scored significantly lower in terms of soft tissue protection. The reason can be attributed to the quality of traditional typodont model. The apparent gap between the resin artificial teeth and silicone gum made it hard to identify the bottom of periodontal pockets, rendering gingiva susceptible to damage. Also, with a larger rigidness and resilience than real gingiva, the tactile sense of silicone gum was different from real mouth cavity. The mimic appearance and deformable features of gingival tissues in VS system helped a lot with mastering the proper scaling force.

Whereas, the fidelity assessment results suggested that there is still room for improvement in the realism of the VS system. As beginners, students were more content with the VS system (Table 1), while experienced teachers found some aspects not realistic enough. These results indicated that VS teaching method might be more suitable for pre-clinical teaching at the moment.

Previous studies had reported that VS technology offers multiple advantages in education, such as increased student confidence and acquisition of manual dexterity skills, as well as achieving good tactile feedback on the cleaning force [24, 25]. Up to now, only few researches have been conducted due to equipment and technology limitation [26]. VS was proved to be helpful in manual supragingival scaling training program [27, 28].

However, the effect of VS technology in ultrasonic scaling teaching program has not been evaluated yet. Ultrasonic scaling differs greatly from manual scaling, not only in the scaler types and means of motion, but also with a much distinct hand feel due to its additional ultrasonic vibration and liquid flushing during entire scaling. The UniDental VS system used in the current research was the first to innovatively meet the pre-clinical education demands [29, 30]. It was equipped with a 6-degree of freedom 3-dimensional force feedback mechanism and had significant technical advantages in real-time (1000 Hz), meticulous force feedback and realistic 3-Dimension scene rendering.

A variety of scaler types were provided with different vibration frequencies, ART P10, P100, P1000 for magnetostrictive and EMS G1 to G6, P1 for piezoelectric, covering supragingival and subgingival scaling. A corresponding vibration pattern of a particular work tip would be preset in the physical-based virtual modeling. And when the force feedback button is turned on, mimetic forcible feedback would be given through handset. The realization of force feedback system involves the following key problems: the designation of force feedback calculation model, collision detection and synchronous simultaneous rendering of vision-force perception. Because the ultrasonic vibration of ultrasonic scalers causes high-frequency collision, the procedure requires extraordinarily high calculating speeds and image-feeling update frame rates, which could be met well by the UniDental VS system. It also provides timely self-assessment for students, which could significantly improve their performance [31].

Despite the outstanding performance of VS in ultrasonic scaling teaching, it is not advised to be used as an alternative to traditional methods due to features such as excessive critical feedback, technical hardware difficulties, lack of personal contact, lack of pain and injury response and relative weaknesses in some perspectives of teaching compared with typodonts or quail eggs [32]. Plessas et al. also reported that guidance and evaluation by professional teachers are indispensable in training courses [33, 34].

The present study proposes an integrative teaching procedure that makes good use of the strengths of different teaching methods. The procedure includes a 15-minute theoretical review, a 30-minute demonstrative teaching on a periodontal pathological typodont, a 45-minute quail egg scraping practice, a 30-minute supra-gingival scaling on a pathological typodont in head-simulators, a 30-minute VS training (supra-gingival), a 45-minute sub-gingival scaling practice on a pathological typodont in head-simulators, and a 45-minute VS training (sub-gingival), followed by a 30-minute operation examination in the pre-clinical stage.

**Fig. 6:**
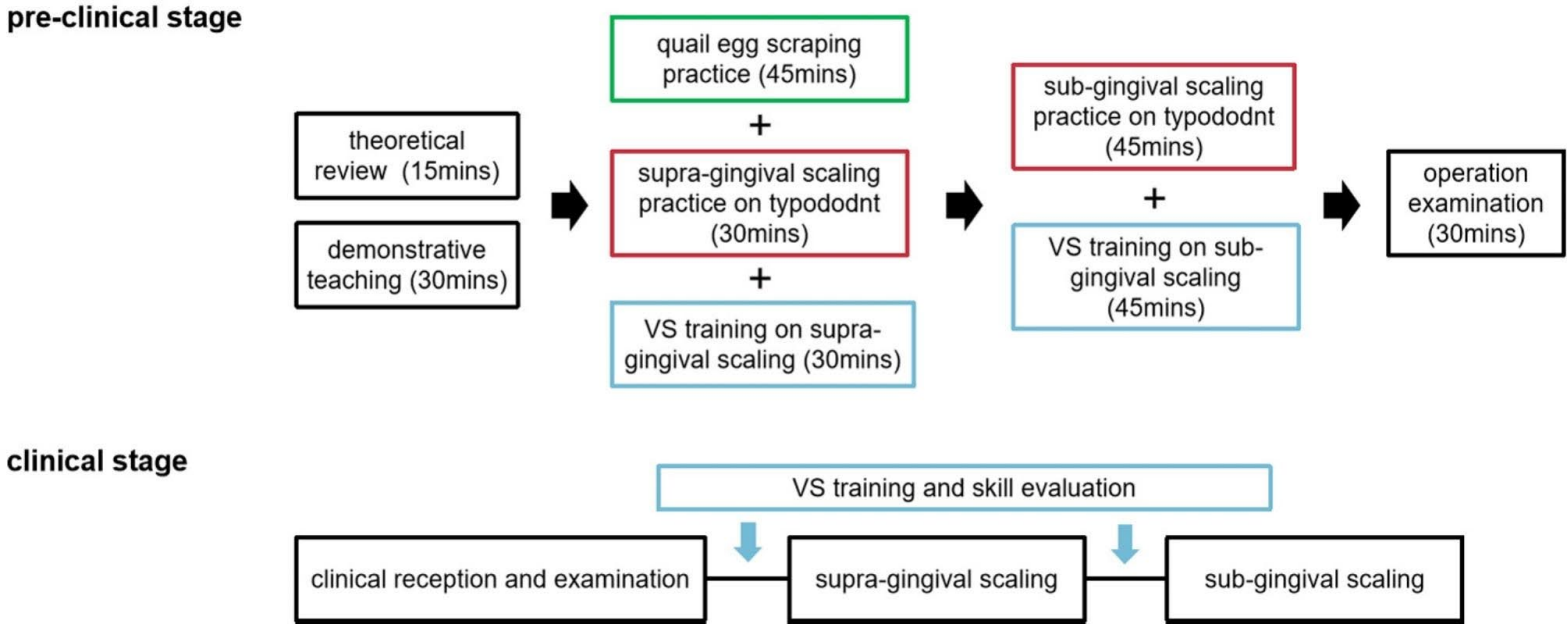
Flow diagram of the recommended periodontal integrated training program

During internship in periodontics, it is recommended to undergo VS training and skill evaluation before supra-gingival scaling and sub-gingival debridement practice in clinic, in order to further improve students’ clinical performances. The specific implementation process is shown in Fig. 6. The combination of VS technology, quail egg, pathological typodont and teachers’ professional guidance could better address the shortcomings of each method, thus promoting theoretical knowledge and acquisition of clinical scaling skills.

This study provides a useful reference for further development of medical teaching models, but it also had several limitations. Firstly, the present study used the calculus residual rates and head-simulator-based assessment to represent student’s training outcomes, instead of direct practical clinical operations on patients, which cast doubt on the representativeness of results. Secondly, the operation scoring and questionnaires had certain subjective bias, attenuating the effectiveness of partial results. Thirdly, the study only accessed short-term effectiveness, which means that the duration of training of the experimental subjects was insufficient to understand the long-term application effects of the different teaching methods. Fourthly, the optimal sequence and proportion for training is still to be further explored. Finally, different manipulation systems of VS may introduce bias, and realistic virtual simulation equipment is necessary for future research.

## Conclusion

In this study, VS technology- and quail egg-assisted ultrasonic training was compared with traditional scaling teaching on pathological typodont from different perspectives. Overall, VS showed the best general performance in teaching as it significantly improved calculus removal efficacy, especially in challenging locations, and helped students to master periodontal scaling techniques effectively. Quail egg was found to be advantageous in force application and supra-gingival scaling, while the typodont was effective for pivot stability practice and body position training. To leverage the strengths of each method, an integrative teaching procedure has been proposed in this study, which aims to provide an effective, advisable and standardized pre-clinical training procedure for ultrasonic scaling.

### Electronic supplementary material

Below is the link to the electronic supplementary material.

**Supplementary Material 1: Supplementary Table 1.** Ultrasonic Periodontal Debridement Evaluation Form. **Supplementary Table 2.** Scoring items of the questionnaire on teaching effects. (Supra-gingival). **Supplementary Table 3.** Scoring items of the questionnaire on teaching effects. (Sub-gingival). **Supplementary Fig. 1** Total sample size estimation by G*Power (version 3.1.9.7). α = 0.05, 1-β = 0.90. The effect size was set according to the means and standard deviation of preliminary experiment (f = 0.52). The sample size was estimated to be more than 51

## Data Availability

The datasets analyzed during the current study are available from the corresponding author on reasonable request.

## Abbreviations

PD: Probing depth
QE group: Quail egg group
SD: Standard deviation
SRP: Scaling and root planing
TT group: Traditional pathological typodont group
VS: Virtual simulation
