# Supplementary material for: Effectiveness of typodont, quail egg and virtual simulation for ultrasonic periodontal scaling teaching among pre-clinical students: a randomized trial
Source: BMC Oral Health. 2024 Jan 16;24:86. doi: 10.1186/s12903-023-03767-5 (PMC10790488; doi:10.1186/s12903-023-03767-5)

Supplementary Table 1. Ultrasonic Periodontal Debridement Evaluation Form

| Name | Student ID | | Age | Gender | Class | | |
| --- | --- | --- | --- | --- | --- | --- | --- |
|  |  | |  |  |  | | |
| Items | | Scoring Standards | | | | Points | Score |
| Dental tools selection | | Supragingival (A) tip (5 points). | | | | 5 |  |
|  |  | Subgingival (P) tip or (PS) tip (5 points). | | | | 5 |  |
| Body position | | Examiner designates tooth position; adjust positioning based on tooth location. Generally, select positions from 8 o'clock to 12 o'clock (2 points).  Sit with legs apart and feet flat on the ground, thighs parallel to the ground, straight waist, shoulders hanging down (4 points).  Maxillary plane to ground plane at a 60-90° angle, mandibular plane at a 0-degree angle (2 points).  Patient's oral cavity aligns with operator's elbow joint (2 points). | | | | 10 |  |
| Periodontal probing | | Use a periodontal probe to examine the depth, location, and shape of the periodontal pocket at six positions around each tooth before scaling (2 points).  Use a pointed probe to examine the location and shape of calculus (2 points). | | | | 4 |  |
| Tools gripping | | Standard relaxed pen grasp or modified pen grasp. | | | | 6 |  |
| Firm pivot | | No fulcrum results in a deduction of 10 points.  Composite fulcrum formed by tight placement of middle finger and ring finger together, or middle finger as fulcrum (4 points).  Finger pad placed on adjacent tooth (4 points).  The fulcrum must remain stable throughout the operation (2 points). | | | | 10 |  |
| Angle of worktip | | 0-15° with the tooth surface.  Deduct points for angles that are too large or too small.  Full deduction for directly aligning the tip with the tooth surface. | | | | 10 |  |
| Generation of strength | | Apply light force to maintain contact with the tooth surface | | | | 4 |  |
| Direction of Force | | Parallel, obliquely upward, or vertically upward.  Full deduction for force directed toward the gingival sulcus. | | | | 10 |  |
| Movement magnitude | | Avoid excessive movement.  Use short-distance, continuous, and variable-direction strokes. | | | | 6 |  |
| Check with probe | | After the operation, check for the presence of supra-gingival and sub-gingival calculus. | | | | 3 |  |
| Calculus removing | | Tooth 31 will be checked by the examiner. (an anterior tooth sample)  Supra-gingival: clean mesial, distal, labial, and lingual surfaces receive 1 point each;  Sub-gingival: clean mesial and distal surfaces receive 2 points each; clean labial and lingual surfaces receive 1 point each. | | | | 10 |  |
|  |  | Tooth 17 will be checked by the examiner. (a posterior tooth sample)  Supra-gingival: clean mesial, distal, buccal, and palatal surfaces receive 1 point each;  Sub-gingival: clean mesial and distal surfaces receive 2 points each; clean buccal and palatal surfaces receive 1 point each. | | | | 10 |  |
| Avoiding soft tissues injury | | Use of a mouth mirror or fingers to protect lip, cheek, and tongue mucosa. Full deduction for gingival tearing. | | | | 7 |  |
| Total | |  | | | |  |  |

Supplementary Table 2. Scoring items of the questionnaire on teaching effects. (Supra-gingival)

| Items | | Points (1-5) |
| --- | --- | --- |
| Mastering the way to fit work tip with tooth surfaces |  | |
| Mastering the fit angle of work tip and tooth surfaces |  | |
| Mastering lateral pressure of scaling |  | |
| Mastering movement and sliding force of scaling |  | |
| Mastering proper grip of tools |  | |
| Keeping proper body position |  | |
| Mastering proper pivots |  | |
| Improvement of scaling efficiency |  | |
| Mastering the proper approaches of work tips |  | |
| Mastering sequence and consistency of supra- and sub-gingival scaling |  | |
| Reducing injuries to tooth tissues in scaling |  | |
| Reducing injuries to gingiva in scaling |  | |

Supplementary Table 3. Scoring items of the questionnaire on teaching effects. (Sub-gingival)

| Items | | Points (1-5) |
| --- | --- | --- |
| Mastering the way to fit work tip with tooth surfaces |  | |
| Mastering the fit angle of work tip and tooth surfaces |  | |
| Mastering lateral pressure of scaling |  | |
| Mastering movement and sliding force of scaling |  | |
| Mastering proper grip of tools |  | |
| Keeping proper body position |  | |
| Mastering proper pivots |  | |
| Improvement of scaling efficiency |  | |
| Mastering the proper approaches of work tips |  | |
| Mastering sequence and consistency of supra- and sub-gingival scaling |  | |
| Reducing injuries to tooth tissues in scaling |  | |
| Reducing injuries to gingiva in scaling |  | |

Supplementary Figure 1


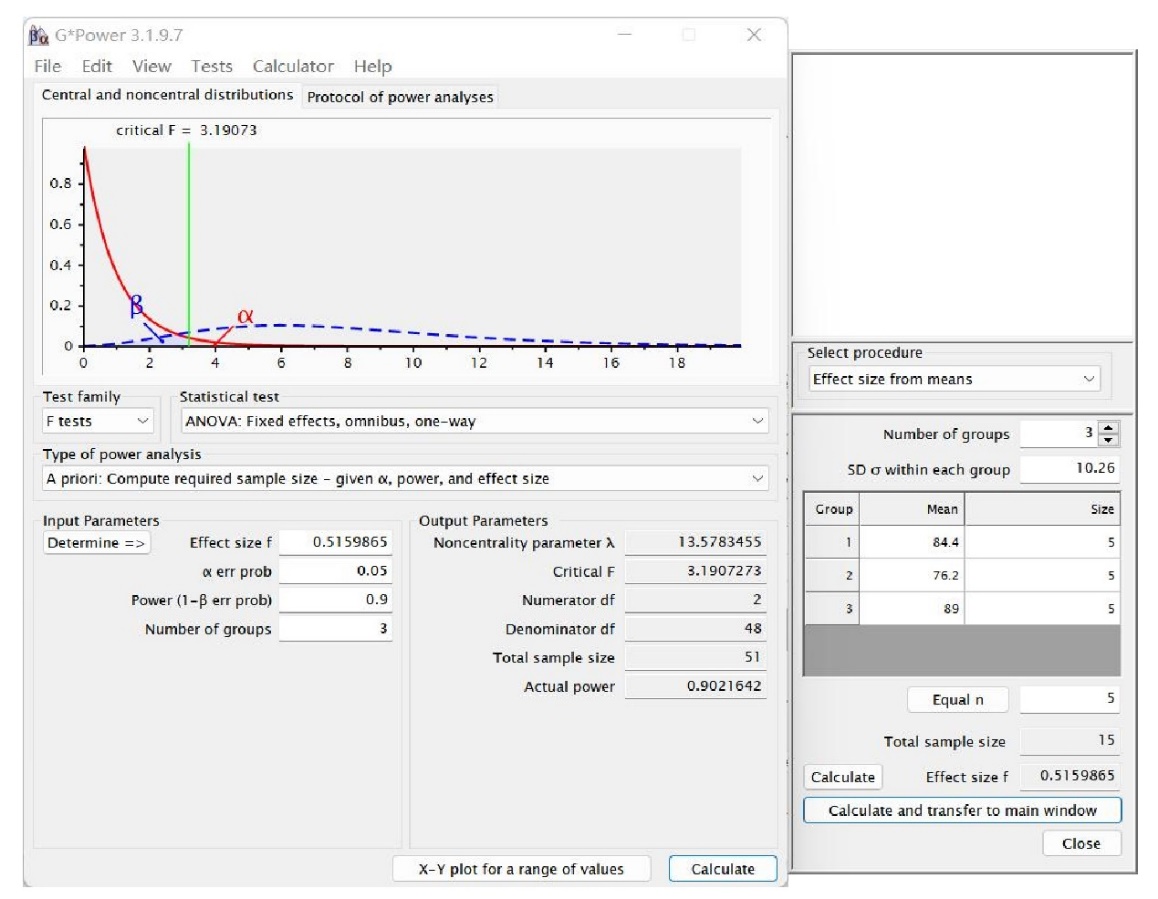

Supplement: Supplementary file 1 — Supplementary Material 1: Supplementary Table 1. Ultrasonic Periodontal Debridement Evaluation Form. Supplementary Table 2. Scoring items of the questionnaire on teaching effects. (Supra-gingival). Supplementary Table 3. Scoring items of the questionnaire on teaching effects. (Sub-gingival). Supplementary Fig. 1 Total sample size estimation by G*Power (version 3.1.9.7). α = 0.05, 1-β = 0.90. The effect size was set according to the means and standard deviation of preliminary experiment (f = 0.52). The sample size was estimated to be more than 51 [file 12903_2023_3767_MOESM1_ESM.docx]
